# Factors influencing participation in colorectal cancer screening among average-risk populations: A descriptive-analytical study in a developing country

**DOI:** 10.1371/journal.pone.0323291

**Published:** 2025-05-23

**Authors:** Farimah Rahimi, Reza Rezayatmand, Zahra Dalvand, Zahra Ravankhah, Peyman Adibi

**Affiliations:** 1 Health Management and Economics Research Center, Isfahan University of Medical Sciences, Isfahan, Iran; 2 School of Management and Medical Information, Isfahan University of Medical Sciences, Isfahan, Iran; 3 Cancer registry of Health Deputy, Isfahan University of Medical Sciences, Isfahan, Iran; 4 Isfahan Gastroenterology and Hepatology Research Center (IGHRC), Isfahan University of Medical Sciences, Isfahan, Iran; Howard University, UNITED STATES OF AMERICA

## Abstract

**Introduction:**

Colorectal cancer (CRC) is a leading cause of cancer-related deaths globally, with early detection significantly improving treatment outcomes. Understanding the factors influencing the decision to participate in colorectal cancer screening programs can help design interventions to improve these programs.

**Method:**

This study investigates factors influencing CRC screening participation among average-risk individuals aged 50–70 years in Isfahan province, utilizing data from the comprehensive E-health information system. Employing Andersen’s Behavioral Model, the study examines predisposing, enabling, and need-for-care factors. A logistic regression model was used to identify significant predictors of screening participation.

**Results:**

The analysis in this study includes 808,978 average-risk individuals in Isfahan, with a mean age of 56.67 years (SD = 7.17). Results indicate that younger age, male gender, marital status, health insurance coverage, flexible work time pattern, Iranian nationality, and high-risk lifestyles positively influence the probability of participation. The predictor screening analysis reveals that lifestyle, chronic disease, and education are the top three factors influencing participation, with lifestyle being the most dominant predictor.

**Conclusion:**

Participation in colorectal cancer screening is influenced by numerous factors. Therefore, it is recommended that all important factors determining individuals’ participation in colorectal cancer diagnosis be considered and that individuals’ lifestyles be adjusted to encourage effective participation in colorectal cancer screening. Understanding these factors can guide targeted interventions to enhance CRC screening uptake and early detection.

## Introduction

Colorectal cancer (CRC) has gained significant attention, becoming the third most prevalent cancer globally in 2022. With 1,926,425 newly diagnosed cases, CRC made up about 10% of all new cancer cases. Even more alarming, CRC ranked as the second leading cause of cancer-related deaths, with 904,019 fatalities, accounting for approximately 9.3% of total cancer deaths[1]. Most CRC cases occur in individuals aged 50 and older, and with the aging global population, the burden of this disease is expected to increase significantly [2].

Screening is a proven strategy for reducing the risk of death from colorectal cancer, and there is compelling evidence indicating that differences in colorectal cancer mortality rates are due to variations in access, utilization, and quality of screening [3]. The American Cancer Society recommends annual Fecal Occult Blood Testing (FOBT) starting at age 50 for the early detection of colorectal cancer in asymptomatic individuals. If the test results are positive, the individual is referred for more precise tests such as colonoscopy and sigmoidoscopy [4,5]. It is believed that most colorectal cancers originate in adenomas, which can be removed to prevent the development of colorectal cancer [6]. Evidence suggests that early detection of adenomas reduces the incidence of this type of cancer. In other words, colorectal cancer screening and early detection can prevent deaths from this cancer [7].

CRC is highly treatable when detected early, with a 5-year survival rate of 90%, but only 39% of cases are diagnosed at this stage, and survival drops to 10% [8]. Despite the effectiveness of screening programs in early disease detection and more effective treatment of colorectal cancer, and given the widespread availability of free or low-cost screening and public education on healthy living, prevention, and early disease detection, many at-risk individuals do not participate in screening programs [9].

Numerous studies have examined factors affecting CRC screening and follow-up adherence[10–34]. A systematic review by Wools et al. (2016), which analyzed 77 studies, identified several significant factors linked to adherence. These included being female, younger age, lower educational attainment, lower income, and belonging to an ethnic minority group [35]. Similarly, in another systematic review, Agunwamba et al. (2023) by using the 5As dimensions found that the most frequent factors were contact with healthcare systems (in Access), insurance (in Affordability), knowledge of colorectal cancer screening (in Awareness), health beliefs (in Acceptance), prompts and reminders (in Activation). Across all reviewed studies, the most common factors identified were healthcare system contact, insurance, race/ethnicity, age, and education [36]. Barriers to CRC screening vary, and their categorization differs as well. However, the Andersen Behavioral Model offers a comprehensive framework by classifying influencing factors into three categories: predisposing, enabling, and need factors [37]. Based on the review by Alkhawaldeh et al (2023), most of the studies examined age, education, gender, marital status, and employment status as predisposing factors, and income, medical insurance, and living location as enabling factors. While, chronic illnesses and perceived general health status were examined as need factors, in addition to an extensive diversity of health conditions and illnesses [38]. Several studies have employed this model to investigate CRC screening uptake. For example, Wong et al. (2022) and Chan et al. (2022) explored factors influencing CRC screening uptake using Andersen’s Behavioral Model in Hong Kong [10,13].

A decade after the launch of the CRC screening program in Isfahan, Iran, no comprehensive study has been conducted to evaluate the factors influencing participation. Therefore, our study aims to investigate the factors associated with participation in CRC screening among average-risk individuals aged 50–70 years in Isfahan province. By identifying these factors, we can better understand the barriers to screening and develop strategies to enhance participation and early detection of CRC.

## Methods

### Study design

This descriptive-analytical study aimed to investigate the factors influencing participation in CRC screening using data from Iran’s Integrated Electronic Health System (SIB, a Persian acronym meaning “apple”). Launched by the Ministry of Health in February 2015, SIB was designed to modernize health data management. The system currently hosts electronic health records (EHRs) for over 73 million individuals and is operational in more than 36,000 urban and rural areas. SIB is distinguished as the most comprehensive and advanced system, offering online functionality and nationwide integration [39].

### Study population

Population in the study had to meet the following inclusion criteria: individuals aged 50–70 years residing in Isfahan province from 2016 to 2021, with information recorded in the SIB system (access date: 5/23/2023). The exclusion criteria were: high-risk individuals for CRC (personal history of cancer, personal history of polyps, IBD, first-degree family history of cancer, second-degree family history of cancer, weight loss, constipation, and bleeding), individuals diagnosed with CRC before the study period, those with no health service records after turning 50, and dead individuals. The study protocol was approved by the ethics committee of Isfahan University of Medical Sciences (IR.MUI.RESEARCH.REC.1398.257). It utilized anonymized secondary data on CRC screening, with all identifying information removed to ensure participant confidentiality. As a result, the need for informed consent was waived by the ethics committee.

### Data collection

We developed a form based on Andersen’s Behavioral Model to address predisposing, enabling, and need-for-care factors, as well as the utilization of CRC screening services, using existing variables. Predisposing factors included age, gender, marital status, and education level. Enabling factors included job, health insurance coverage, and nationality. The need-for-care factors comprised comorbidities and lifestyle factors. Comorbidities included hypertension, hyperlipidemia, diabetes, and cardiovascular diseases. Lifestyle factors included physical activity level, obesity, and consumption of salt, oil, vegetables, fruits, and fast food. In this study, the lifestyle variable was used to categorize individuals into three groups: those with the highest scores were classified as the “healthiest group,” those with the lowest scores as the “high-risk group,” and those with intermediate scores as the “low-risk group.”

### Statistical analysis

The analysis utilized a logistic regression model to identify factors associated with participation in colorectal cancer (CRC) screening. The likelihood of participation was estimated using a logit model, taking into account predisposing, enabling, and need factors. Data were analyzed using SAS JMP Pro 17. Variables with p < 0.001 were selected as candidate independent variables for multivariable logistic regression analysis. The goodness-of-fit for the multivariable logistic regression models was assessed using the Whole Model Test, Wald test, and Lack of Fit test. Results were presented as odds ratios (OR) with their corresponding 95% confidence intervals (CI) at a significance level of 0.001. Additionally, probabilities were presented based on the model, and predictor screening was performed using 100 bootstrapping iterations. Predictor Screening is a method used to evaluate and rank predictors based on their ability to explain the variability in the outcome variable, which in this case is participation. The contribution represents the total impact of the predictor on the model’s ability to predict participation. A higher value indicates a stronger influence on the prediction accuracy. Portion is the proportion of the total contribution made by each predictor. This gives a sense of the relative importance of each predictor in the model. Predictors are ranked based on their contribution, with 1 being the most significant predictor.

## Results

### Individuals’ characteristics

A summary of the characteristics of 808,978 average-risk individuals in Isfahan, with a mean age of 56.67 years (SD = 7.17), is presented in Table 1. Over half of the individuals were men, most had elementary education, 87.7% were married, and 97.72% were Iranian. More than half do not have the disease and less than half have a healthy lifestyle. More details are presented in Table 1.

**Table 1:** Average Risk Individuals’ characteristics (N = 808978).

Variable	Description	Count	Percentage
Sex	Men	434,750	0.53
	Women	374,228	0.46
Age	≤ 65 years	689,899	0.85
	> 65 years	119,081	0.15
Marital Status	Married	709,195	0.87
	Single	99,410	0.12
Insurance Status	Insured	770,444	0.95
	Uninsured	38,534	0.047
Work Time Pattern	Free	433,742	0.53
	Flexible	259,469	0.32
	Fixed	115,767	0.14
Nationality	Iranian	790,540	0.97
	Non-Iranian	18,438	0.02
Education	Illiterate	131,955	0.16
	Elementary	263,185	0.33
	Diploma	306,950	0.38
	Bachelor’s	89,059	0.11
	Master’s	12,140	0.015
	Above	5,690	0.007
Comorbidities	None	542,430	0.67
	1 disease	142,365	0.18
	2 diseases	79,679	0.098
	3 and more diseases	44,505	0.054
Lifestyle	Healthy	271,391	0.48
	Low risk	212,673	0.37
	High risk	86,250	0.15

Over the five years of the screening program, approximately 40% of individuals participated in colorectal cancer screening.

### Logit regression

The logistic regression analysis examined the factors influencing participation. The odds ratios (ORs) for changes in the levels of independent variables are presented in Table 2:

**Table 2:** Odds Ratios for changes in the levels of independent variables.

Variable (reference group)	Level	Odds Ratio	Prob>Chisq	Lower	Upper
**Education**		0.919513	<.0001*	0.918286	0.92074
**Chronic Disease (**None)	1	1.9720767	<.0001*	1.944901	1.9996321
	2	2.5214252	<.0001*	2.4759485	2.5677372
	>3	2.9826452	<.0001*	2.9107287	3.0563386
**Age Group (**<65)	>65	0.3882414	<.0001*	0.3808139	0.3958136
**Lifestyle (**healthy)	Low-risk	0.9908289	0.2433	0.9756135	1.0062816
	High-risk	1.848898	<.0001*	1.8214279	1.8767825
**Marital status (**Single)	Couple	1.1577514	<.0001*	1.1370209	1.1788598
**Nationality (**iranian)	noniranian	0.693506	<.0001*	0.6577703	0.7311832
**Work Time Pattern(**free)	fix	0.784342	<.0001*	0.7706882	0.7982377
	flexible	0.8128618	<.0001*	0.7957546	0.8303368
**Sex (**Male)	Female	0.8956214	<.0001*	0.880516	0.9109859
**Insurance (**No)	Yes	1.8519251	<.0001*	1.7792193	1.927602

### Predisposing factors

The study considered several predisposing factors, including age, gender, marital status, and education level. Age also had a notable effect, with individuals over 65 having significantly lower odds of participation compared to those younger than 65 (Odds Ratio = 0.388, p < 0.0001). Gender also played a role, as males had higher odds of participation compared to females (Odds Ratio = 0.896 for females, p < 0.0001). Marital status also affected participation, as being part of a couple increased the odds of participation compared to being single (OR = 1.158, p < 0.0001). Education had a slightly negative effect on participation, with a per-unit increase in education reducing the odds of participation (Odds Ratio = 0.919, p < 0.0001).

### Enabling factors

Enabling factors, such as employment status, health insurance coverage, and nationality, were also significant. Work Time Pattern also influenced participation, with those having flexible or freelance times being more likely to participate compared to those with fixed or flexible times (fixed vs. free: Odds Ratio = 0.784, p = 0.0001; flexible vs. free: Odds Ratio = 0.813, p < 0.0001). Health insurance coverage was another key enabling factor. Individuals without health insurance were much less likely to participate compared to those with insurance, while individuals with insurance were almost twice as likely to participate (Odds Ratio = 1.852, p < 0.0001). Non-Iranians had significantly lower odds of participating compared to Iranians (Odds Ratio = 0.694, p < 0.0001).

### Need factors

Need factors, which include the presence of chronic diseases and lifestyle factors, were among the most influential. The presence of chronic diseases increased the odds of participation, with a gradient effect observed: One chronic disease (OR = 1.972, p < 0.0001), two chronic diseases (OR = 2.521, p < 0.0001), and more than three chronic diseases (OR = 2.983, p < 0.0001). Individuals with high-risk lifestyles were significantly more likely to participate compared to those with healthy lifestyles (OR = 1.849, p < 0.0001) but low-risk lifestyle participants had similar odds to healthy individuals (OR = 0.991, p = 0.2433).

### Model fit

The model demonstrated a good fit based on the Whole Model Test. Key fit statistics included a -LogLikelihood Difference of 34,158.13 and a Chi-Square value of 68,316.27 with a p-value less than 0.0001. The results of the lack of fit test show a Chi-Square value of 11,088.52 and a p-value less than 0.0001, indicating a significant lack of fit. Effect Wald Tests for participation indicate several significant predictors. The key effects, ranked by their significance (Logworth), include disease, education, age, lifestyle, insurance, job type, marital status, nationality, and sex, all with a p-value of 0.00000, demonstrating highly significant impacts on participation.

### Probability

Gender differences are also observed, as females exhibit a lower probability of participation compared to males. The probability of Participation is greater for the Age Group=>65 than <65. Marital status impacts participation, with a couple of individuals having a slightly higher probability of participation compared to those in singles. Furthermore, insurance coverage positively influences participation; individuals without insurance have a lower probability of participation, whereas having insurance increases the probability. Individuals with fixed or flexible job types have a lower participation probability, while those with free job types show higher participation probabilities. Education level also affects participation, with a slight but noticeable decline in participation probability as education level increases. This suggests that individuals with higher education levels tend to participate less. Nationality is another influential factor, with Iranians showing a higher participation probability compared to non-Iranians. Lifestyle is a significant determinant as well; healthy individuals have the lowest participation probability, which increases for individuals with low-risk lifestyles and further increases for those with high-risk lifestyles.

**Fig 1:**
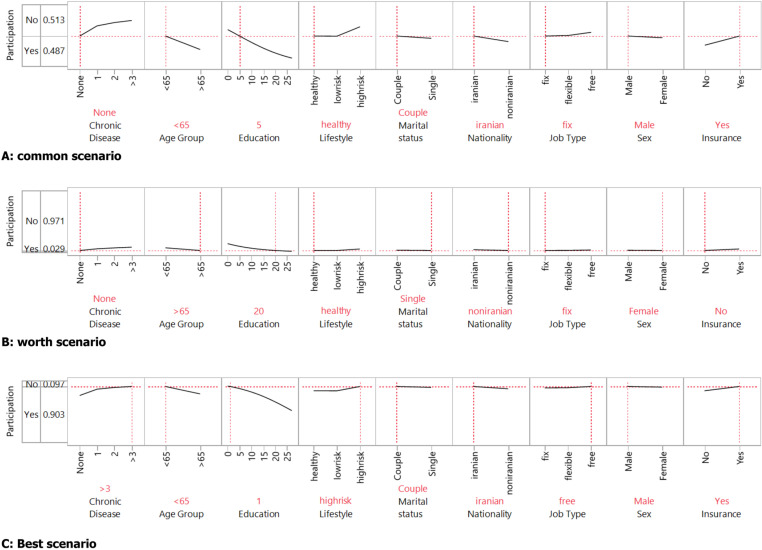
Prediction Profiler: Effects of Predictors on CRC Screening Participation Probabilities. A: Common Scenario – Displays actual predictor values and their associated participation probability. B: Worst-case Scenario – Demonstrates the lowest probability of participation (3%) under unfavorable predictor conditions. C: Best-case Scenario – Shows the highest probability of participation (90%) under optimal predictor conditions.

Fig 1, visually represents the effects of various predictors on participation probabilities from the logistic regression analysis. This provides a comprehensive overview of how each factor affects participation, aligning with the logistic regression analysis results. Understanding these effects can guide policy-making and interventions aimed at improving participation rates. This visual summary presents common scenarios, including the best and worst-case scenarios, with probabilities of participation at 3% and 90%, respectively.

### Predict participation

Table 3 summarizes the contribution and importance of each predictor in determining participation. The predictor screening analysis reveals that lifestyle, chronic disease, and education are the top three factors influencing participation, with lifestyle being the most dominant predictor. This information can be used to target interventions and policies more effectively, prioritizing changes in lifestyle and health management to enhance participation rates.

**Table 3:** Relative Contribution and Ranking of Predictors Influencing Participation.

Predictor	Contribution	Portion	Rank
Lifestyle	129316	0.6615	1
Chronic Disease	40442	0.2069	2
Education	17507	0.0896	3
Age Group	3725	0.0191	4
Job Type	1617	0.0083	5
Insurance	1285	0.0066	6
Sex	1128	0.0058	7
Nationality	328	0.0017	8
Marital status	135	0.0007	9

## Discussion

Colorectal cancer (CRC) screening is crucial for early detection and reducing mortality. Despite being offered free-of-charge under the IraPEN program, only about 40% of the target population chooses to participate. This study aimed to analyze factors influencing CRC screening participation among average-risk individuals in Isfahan, using a large sample of over 800,000 people.

Our analysis identified a range of predisposing, enabling, and need factors influencing participation in CRC screening. Some of these findings are consistent with prior research, while others highlight unique patterns within the population studied. These results underscore the importance of contextual influences and provide a foundation for tailoring interventions aimed at improving screening rates. By addressing the gap in understanding real-world screening behavior, this study makes a meaningful contribution to the field and highlights areas for further exploration in prospective research.

### Gender:

Our study found that males had a higher probability of participating in CRC screening than females with other variables in the model. This is in line with findings from studies that reported higher CRC screening rates among men compared to women[10–12]. The reasons for this may be linked to women’s reported discomfort or fear about the screening process [12]. However, some studies show opposite results, found no significant gender differences in CRC screening uptake [13], or found Overall screening uptake is higher in women than men [14]. Thus, barriers to CRC screening may differ by sex [15], This discrepancy may be due to cultural or healthcare system differences, suggesting that gender-related participation might be more context-specific.

### Age:

Our study confirmed that younger adults had higher participation rates, aligning with the findings of Koo et al. (2012) in the Asia-Pacific region. Koo’s study indicated that respondents aged 50 and above from Hong Kong, Indonesia, Korea, and Taiwan exhibited lower awareness of symptoms, risk factors, and, to a lesser extent, screening tests compared to their younger counterparts. However, no significant age-related differences were observed in the other countries studied [16]. Chan et al. (2022), observed that younger individuals were more likely to participate in colorectal cancer screening [13]. However In contrast, Huang et al. (2021) reported that older individuals (aged 66–70) were more likely to participate, potentially due to targeted awareness campaigns within those populations [17]. This highlights the variability in age-related participation rates depending on public health strategies and underscores the need for interventions aimed at older age groups.

### Marital status:

Marital status Marital status plays a significant role in influencing participation in health screenings. Research by Gram et al. (2020) found a strong link between marital status and CRC screening, with participation rates being particularly high among married individuals, especially those whose partners also participated [18]. Similarly, El-Haddad et al. (2015) reported that in the USA, both married individuals and those in unmarried partnerships had higher odds of undergoing CRC screening compared to other marital status groups[19]. Chan et al. (2022) observed comparable trends in multi-ethnic Asian populations, where married people were more likely to engage in health services, including CRC screening [13].

### Education:

Interestingly, in our study, higher education was linked to lower participation in screening (OR = 0.920), which challenges the common belief that greater education enhances health literacy and preventive care behaviors. Willems and Bracke (2018) found that individuals with higher education levels were more likely to participate in screenings for cervical, breast, and colorectal cancer compared to those with lower education levels. However, they also noted that educational disparities were smaller in countries with organized cervical cancer screening programs than in those with opportunistic screening[20]. Budhraja et al. (2011) highlighted that patient education is a powerful but underutilized tool for improving the effectiveness of CRC screening[21]. These contrasting results suggest that while education typically boosts health literacy, its impact on screening participation may vary based on factors such as trust in healthcare systems and access to screening services. Our findings indicate that individuals with higher education levels participated less in the screening program. The mean years of education in the study population was approximately 8 years (SD = 4.89), suggesting a relatively low overall education level. However, since the screening program was conducted within the healthcare system and primarily targeted rural populations, participation was naturally higher in rural areas compared to urban regions. Additionally, as these services were provided during standard working hours, individuals with higher education—who are more likely to be employed in fixed-hour jobs—may have faced more barriers to participation. Another possible explanation is that trust in the healthcare system may be lower among more educated individuals, influencing their willingness to engage in such programs. Given these factors, the observed results are not unexpected.

### Nationality:

Our results indicated that Iranian nationality was positively associated with higher participation in colorectal cancer (CRC) screening (OR = 1.442). Warren Andersen et al. (2019) found lower CRC screening rates among African American individuals and those with low socioeconomic status[22]. Similarly, a study in Norway revealed that immigrants, particularly from non-Western countries, participated less frequently in a CRC screening pilot compared to non-immigrants [23]. This lower participation among immigrants may be attributed to factors such as language barriers, cultural beliefs, and limited awareness about screening programs.

### Work time pattern:

Employment type influenced CRC screening participation in our study, with individuals in flexible or freelance work time patterns showing higher participation rates. Similarly, Fedewa et al. (2022) reported that unemployment was linked to lower cancer screening rates, largely due to a lack of health insurance[24]. Collatuzzo et al. (2022) highlighted that cancer disparities are partly rooted in occupational factors such as job type, position, tasks, work schedule, salary, and employment status. These factors contribute to varying cancer screening participation across different working categories, reflecting disparities in access to health services[25].

### Insurance:

Individuals with health insurance are more likely to pursue preventive care, including CRC screening, among U.S. adults[26]. Gawron et al. (2021) noted that the lack of insurance coverage and the high cost of care were significant barriers to CRC screening[27]. However, in a study by Jones et al. (2022), health insurance status was not found to be associated with the earlier stage of diagnosis or the mode of detection in a diverse group of patients recently diagnosed with colon cancer[28]. This suggests that while insurance influences screening participation, it may not necessarily impact the stage at which cancer is detected.

### Chronvic diseases:

Our study found that individuals with chronic diseases were more likely to participate in CRC screening. Coronado et al. (2022) observed an inverted U-shaped relationship between a patient’s chronic disease burden and a provider’s recommendation for a FIT and a negative linear relationship between chronic disease burden and FIT completion[29]. Similarly, Guiriguet et al. (2017) found that having three or more major chronic conditions was associated with lower participation in FIT-based CRC screening programs, while individuals with several minor chronic conditions were more likely to participate[30]. Bhatia et al. (2021) highlighted that chronic comorbidities can act as barriers to periodic, guideline-recommended CRC testing, indicating the need to explore cancer prevention gaps in these populations[31]. This discrepancy could be attributed to differences in the implementation of screening programs, where individuals with chronic diseases may have easier access to preventive services.

### Lifestyle:

In our study, individuals with high-risk lifestyles were more likely to participate in CRC screening compared to those with healthier lifestyles. Knudsen et al. (2022) observed a trend of inconsistent participation among individuals with lower healthy lifestyle scores[32]. Carey and El-Zaemey (2019) found that in Australia, CRC screening participation was higher among those practicing healthier behaviors, suggesting that lifestyle patterns may play a key role in screening decisions[33]. Mertens et al. (2024) emphasized that identifying high-risk groups based on lifestyle data can help promote participation in both lifestyle changes and screening programs, thereby reducing the CRC burden. They also advocated for incorporating lifestyle predictors into public health models to enhance screening uptake[34].

Two studies employing Anderson’s model in the context of colorectal cancer (CRC) screening are those by Jin et al. (2019) and Chan et al. (2022). In the study by Jin et al. (2019), researchers examined Korean Americans (KAs) and found low adherence to CRC screening. They identified several predisposing and enabling factors influencing participation, such as age, household income, health insurance, regular health check-ups, doctors’ recommendations, English proficiency, CRC screening knowledge, self-efficacy, and decisional balance. Notably, enabling factors like income and doctor recommendations were predictive of adherence to screening guidelines among KAs [13]. Conversely, Chan et al. (2022) explored individual and contextual factors affecting the uptake of fecal occult blood tests (FOBT) and colonoscopy in Hong Kong. They highlighted government subsidies as the most significant enabling factor for screening participation. Additionally, perceived barriers to screening emerged as a crucial predisposing factor hindering uptake. The authors emphasized the need for ongoing promotion of subsidized screening programs and the development of targeted educational materials to address these barriers[38].

While the cross-sectional design of this study limits our ability to establish causation, it provided a practical and efficient way to analyze a large volume of real-world data. Despite this limitation, the breadth of the data allowed us to identify critical trends and associations that offer valuable insights into screening participation. These findings not only contribute to the existing body of knowledge but also lay the groundwork for future studies with more rigorous designs, such as prospective cohort studies.

## Conclusion

This study highlights critical factors affecting participation in colorectal cancer screening among average-risk individuals in Isfahan province. The profile of participants based on other studies is consistent with our findings. Our study revealed that younger age, male gender, marital status, health insurance coverage, flexible employment, Iranian nationality, and high-risk lifestyles significantly increased CRC screening participation. In contrast, higher education levels, fixed job types, and chronic diseases were associated with decreased participation. These insights suggest that tailored strategies are needed to address barriers in various demographic groups to improve overall screening rates.

### Limitations and recommendations for future research

This study has several limitations. The use of registered data may introduce reporting biases. The generalizability of the findings may be limited to the population registered in the SIB system, and there may be other unmeasured factors influencing participation in colorectal cancer screening. Future research should explore the underlying reasons for the observed differences in screening participation by predisposing, enabling, and need factors level. Longitudinal studies could provide deeper insights into how changes in health policies and educational campaigns impact screening rates over time.

## Declarations:

**Consent for publication**

Not applicable.
